# Analyzing the Role of Fe^0^ and Fe^3+^ in the Formation of Expanded Clay Aggregates

**DOI:** 10.3390/ma16165623

**Published:** 2023-08-14

**Authors:** José Manuel Moreno-Maroto, Beatriz González-Corrochano, Ana M. Martínez-Rodríguez, Antonio Conde-Sánchez, Carlos Javier Cobo-Ceacero, Jacinto Alonso-Azcárate, Manuel Uceda-Rodríguez, Ana B. López, Carmen Martínez-García, Teresa Cotes-Palomino

**Affiliations:** 1 Department of Geology and Geochemistry, Faculty of Sciences, Autonomous University of Madrid, Cantoblanco, 28049 Madrid, Spain; 2 Department of Chemical, Environmental and Materials Engineering, Higher Polytechnic School of Linares, Scientific and Technological Campus of Linares, University of Jaén, 23700 Linares, Spain; bcorroch@ujaen.es (B.G.-C.); cjcobo@ujaen.es (C.J.C.-C.); muceda@ujaen.es (M.U.-R.); ablopez@ujaen.es (A.B.L.); cmartin@ujaen.es (C.M.-G.); mtcotes@ujaen.es (T.C.-P.); 3 Department of Statistics and Operations Research, Campus of Las Lagunillas, University of Jaén, 23071 Jaén, Spain; ammartin@ujaen.es (A.M.M.-R.); aconde@ujaen.es (A.C.-S.); 4 Department of Physical Chemistry, Faculty of Environmental Sciences and Biochemistry, University of Castilla-La Mancha, Avenida Carlos III, s/n, 45071 Toledo, Spain; jacinto.alonso@uclm.es

**Keywords:** expanded clay aggregate, kaolin, iron, organic carbon, sodium carbonate, design of experiments

## Abstract

The effect of the addition of Fe^0^ and Fe^3+^ on the formation of expanded clay aggregates was studied using iron-free kaolin as an aluminosilicates source. Likewise, the incorporation of cork powder as a source of organic carbon and Na_2_CO_3_ as a flux in the mixtures was investigated in order to assess its effect in combination with the iron phases. An experimental protocol, statistically supported by a mixture experiments/design of experiments approach, was applied to model and optimize the bloating index, density, absorption capacity, and mechanical strength. The process of expansion and pore generation and the associated decrease in density required the addition of iron, such that the optimum mixtures of these properties presented between 25 and 40 wt.% of Fe^0^ or Fe^3+^, as well as the incorporation of 3.5–5 wt.% of organic carbon. The addition of Fe^3+^ produced a greater volumetric expansion (max. 53%) than Fe^0^ (max. 8%), suggesting that the formation of the FeO leading to this phenomenon would require reducing and oxidizing conditions in the former and the latter, respectively. The experimental and model-estimated results are in good agreement, especially in the aggregates containing Fe^0^. This reinforces the application of statistical methods for future investigations.

## 1. Introduction

The environmental challenges that humanity is currently facing are leading to changes in production paradigms at a global level, and the construction and civil engineering sectors are no exceptions. Thus, a great deal of research is focusing on the development of building materials that are not only sustainable in their production, but also environmentally advantageous once implemented in practice [1]. In this sense, lightweight aggregates are candidates to consider, since they can be manufactured from a wide range of wastes [2]. In addition, highly porous lightweight aggregates provide much lower thermal conductivity than conventional aggregate construction elements, which translates into a substantial reduction in the energy consumption of the buildings that contain such lightweight aggregates [1,2,3]. 

It is essential to know which raw materials, and in what proportions, are suitable for manufacturing lightweight aggregates. The use of clays as a mineral base to produce expanded lightweight aggregates (i.e., expanded clay aggregates) is very common, together with the use of other raw materials, such as shale and slate [4]. However, in many cases, the clay itself is not capable of expanding when fired (usually for a few minutes in a rotary kiln), due to the absence or inadequate quantities of certain components. Iron seems to be one of the essential elements in this respect. 

The existing literature on how iron affects the expansive process of lightweight aggregates is, on the one hand, somewhat scarce and, on the other hand, very dependent on the particularities of the samples studied and the test conditions, requiring further investigation. One of the earliest studies on this subject was that of Toyama and Kawamura [5], who took some important steps in understanding the role of iron in the formation of lightweight aggregates. Starting with a sample containing 4.4% Fe_2_O_3_, they studied, in detail, the kinetics of the reactions leading to the porous structure of the lightweight aggregates. They concluded that the redox conditions varied in the profile of the ceramic body, so that in the shell, the oxidation state of iron tended to be Fe^3+^, while in the core the reduced form, Fe^2+^, played a crucial role in the process of expansion and pore generation. The latter process was linked to the development of a mineral matrix with adequate viscosity to trap gases, such as the O_2_ released from the iron-reduction reactions. 

Sandrolini and Palmonari [6] found similar results using mixtures with kaolin, calcium carbonate, and 6 wt.% Fe_2_O_3_, highlighting the role of iron as both a flux and a potential gas-releasing agent. More recently, Bernhardt et al. [7] evaluated the impact of adding 5 wt.% of both Fe (as zero-valent iron powder) and Fe_2_O_3_ to clay, along with Na_2_CO_3_ and SiO_2_. The results showed that both iron phases enhanced the bloating process, which was linked to pore formation in the lightweight aggregate. Wie et al. [8] investigated clay mixtures with different percentages of activated carbon and Fe_2_O_3_. Again, the results indicated that Fe_2_O_3_ reduction appeared to be a key factor in producing the lightweight aggregate, in which case it was necessary to add more than 5 wt.% of iron along with percentages higher than 2 wt.% of activated carbon. However, not all researchers have reached the same conclusions as those described above; in some cases, they found that iron oxidation reactions were clearly predominant in the formation of lightweight aggregates, in comparison with reduction reactions [9,10].

The present investigation aims to explore the role of iron in the formation of lightweight aggregates. The work published by Moreno-Maroto et al. [11] focused on the study of pyrite (FeS_2_) as a source of iron in mixtures with kaolin. In addition, the incorporation of two other additives, cork powder as a source of organic carbon and Na_2_CO_3_ as a flux, was investigated. A statistical approach based on the mixture experiments/design of experiments (ME-DOE) method was applied. This approach led to much more powerful protocols than those used to date for understanding the formation process of lightweight aggregates in mixtures with iron. 

The application of statistical methods in the development of ceramic materials has been previously tested, successfully, by other authors who used approaches that included mixture experiments [12], response surface methodology, the fuzzy synthetic evaluation algorithm, second-order polynomial models [13,14], uniform design [15], and machine learning with linear regression, random forest, and support vector regression [16]. The work of Moreno-Maroto et al. [11] corroborated the suitability of ME-DOE in the manufacture of ceramic aggregates, since such techniques had not been previously employed for these types of materials. In mixtures containing pyrite (FeS_2_), the results showed that it did not promote the bloating process of the aggregates they studied; in fact, their findings showed the opposite. Likewise, this phenomenon was not favored with the addition of organic carbon or sodium carbonate. 

The study presented here is based on the same methodology; however, in this case, the main objective was to elucidate the effect of Fe and Fe_2_O_3_ on the synthesis of expanded clay aggregates. Furthermore, we intended to demonstrate that the presence of iron, in combination with other additives (e.g., organic carbon), is essential in synthesizing lightweight aggregates. This study focuses on optimizing the mixture composition best suited to producing lightweight, expanded clay aggregates.

## 2. Materials and Methods

### 2.1. Raw Materials: Clay and Additives

The kaolin used in the mixtures, K, was supplied by Caobar, S.A. (Taracena, Spain) and, based on laser diffraction results, presents an average particle size of 8.3 µm and a d_50_ of 4.6 µm (i.e., 50% of K particles are smaller than 4.6 µm). According to X-ray diffraction and TOC-analyzer tests, K is a clay rich in kaolinite, free of organic matter and carbonates, and with minor amounts of quartz and illite. An X-ray fluorescence analysis has revealed that its chemical composition is rich mainly in SiO_2_ and Al_2_O_3_ (51 and 35%, respectively). Other elements such as alkali, alkaline-earth metals and iron are hardly present [11]. This is very positive, as it allows their proportion to be controlled through the incorporation of the additives detailed below.

Two iron sources with different degrees of oxidation have been selected, Fe^0^ and Fe^3+^, both of which are commercialized by Scharlau^®^. In the case of the former, extra-pure, zero-valent, metallic iron, Fe, was employed (hereafter, I), while Fe_2_O_3_ (hematite; hereafter H), synthesis grade, was used for the latter. Both were in powder form with a particle size of less than 100 µm. Their thermal behavior has been studied via Differential Thermal Analysis Thermogravimetric Analysis (DTA-TG; equipment: SETARAM^®^) at 10 °C/min up to 1200 °C, with air and Ar flow rates of 30 and 40 mL/min, respectively. In this study, the DTA-TG test helps us to understand the transformations undergone by the various components of the mixtures during heating.

The other additives were sodium carbonate (N) and cork powder (C). N is sodium carbonate anhydrous for analysis (PanReac^®^), which would act as a flux component. In addition, the source of organic carbon, C, was <500 µm recycled cork powder (Eurotapón Núñez, S.L., San Vicente de Alcántara, Spain) with a calorific value of ~3 × 10^4^ kJ/kg [11]. 

### 2.2. Mixture Design

The design of mixtures has been carried out with the software R version 4.3.0 (*mixexp* package), so four components have been considered in both the Fe^0^ and Fe^3+^ studies: K, N, C and the iron source, I or H, respectively. The experimental designs are based on the constrained mixture designs. An extended extreme vertex design with centroids was implemented using the *Xvert* and *Fillv* functions of the R package *mixexp* and the *optFederov* function of the *AlgDesign* package. In order to fit the more complex model, these functions replicate certain mixtures.

Based on a previous study [11], the range of C and N percentages was set between 0 and 5 wt.%, since higher values could negatively affect the operability of the tests and the technological properties to be obtained. Regarding the iron additives, the five percentages studied were 0, 12.5, 25, 37.5 and 50 wt.%. The establishment of these percentages was based on tentative preliminary tests, which suggested that the behavioral trends of the material could be investigated in this range. For its part, the kaolin content ranged from 40 to 100 wt.%. Thus, a total of 36 mixtures have been obtained for each iron additive (Table 1), of which the first ten formulations do not contain any iron phase, which will allow a more rigorous understanding of the impact of I and H.

### 2.3. Manufacture and Characterization of Sintered Aggregates

The components of the mixtures were dry blended, and after it was verified that the plasticity was maintained at values suitable for extrusion and granulation (Plasticity Index/Liquid Limit ratios between approx. 0.4 and 0.5 in all mixtures), the optimum moisture content was calculated as 1.495 × Plastic Limit, and this was added during preparation [11,17], with values in the range of ~30 ± 10% of added water. These figures were in agreement with those obtained by the authors in mixtures containing K, N, and C with ground pyrite (FeS_2_) [11]. The wet mixtures were hermetically sealed for 24 h, and then mechanically extruded to facilitate subsequent shaping into ~9.3 mm-diameter round pellets. After drying first in the air and then in an oven at 105 °C, the specimens were subjected to heat treatment in a Nannetti^®^ TOR-R 120-14 rotary tube kiln. During the first 2 min, the specimens remained in the first section of the tube to facilitate preheating and avoid breakage of the material due to thermal shock. Then, the granules were fired for 10 min at their maximum operational temperature, at which the material could experience its largest bloating (if any) and degree of sintering. In addition, exceeding this temperature would cause the granules to stick to each other and to the tube wall. The aggregates obtained were characterized, determining their main technological characteristics, particularly those related to the volumetric expansion experienced (bloating index), density, porosity, water absorption capacity and mechanical strength (single-aggregate crushing test). The specific parameters determined, together with the equipment and test methods, are shown in Table 2.

### 2.4. Modelling and Optimization

Previous work [11] has demonstrated that the application of a statistical approach based on ME-DOE facilitates: (i) preparing a feasible number of starting samples; (ii) studying the synergistic effects of the different components involved during heating; and (iii) modeling to subsequently optimize the mixtures and desired properties. 

Of the properties listed in Table 2, this paper has focused on an ME-DOE statistical analysis of bloating index (*BI*), oven dry density (*ρ*_rd_), water absorption (*WA*_24_) and crushing strength (*S*) through the software R, following the same methodology as that described by Moreno-Maroto et al. [11]. Cubic, special cubic, quadratic, and linear regression models have been compared, and the most suitable selected based on different diagnostic tests. In order to determine the impact of each component of the mixture, effect plots have been obtained. These graphs illustrate how the response changes as the proportion of one component increases or decreases with respect to the centroid (located in this study at K = 70 wt.%, I or H = 25% wt.%, C = 2.5 wt.% and N = 2.5 wt.%) while maintaining the proportion between the other three components (the so-called *Cox directions*). These trends have also been supported with contour plots, which have not been included in this paper so as not to exceed the number of figures. For optimization, the maximum possible results of *BI*, *WA*_24_ and *S* have been considered as targets, applying the opposite criterion for *ρ*_rd_. Finally, the theoretically optimal aggregates estimated for each parameter have been prepared and characterized in the laboratory following the same procedures as those described in Section 2.3. The estimated and experimental results were compared with each other to check the validity of the models.

## 3. Results and Discussion

### 3.1. LOI and Working Temperature

**Figure 1:**
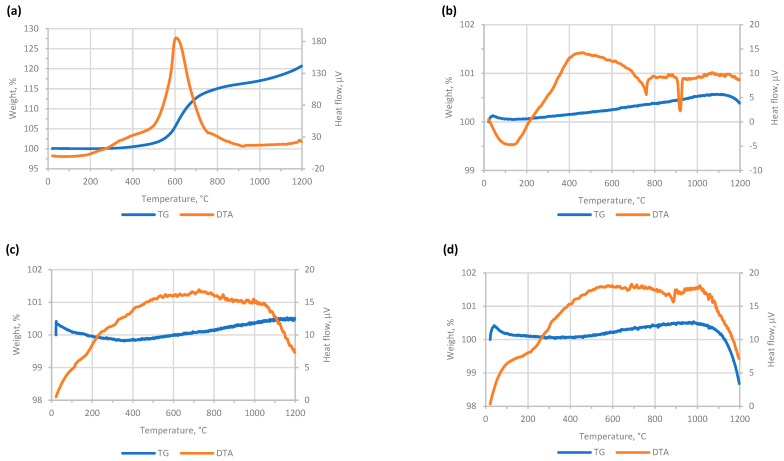
DTA-TG plots in both air and argon atmosphere for the iron additives studied in the mixtures: (**a**) I in air; (**b**) I in Ar; (**c**) H in air; (**d**) H in Ar; where I and H are Fe and Fe_2_O_3_, respectively.

The dataset relating to the characteristics of the aggregates initially obtained is shown in Tables S1 and S2, in the Supplementary Materials. First of all, comparing the LOI during preheating versus that during full firing, an anomalous behavior is observed in the case of thirteen samples containing native iron (Table S1). The aggregates from mixtures I11, I13, I15, I17, I21, I24, I25, I27, I28, I30, I31, I34 and I36 show a lower total mass loss than that observed during preheating. This phenomenon could be attributed to the iron gaining mass through oxidation in the kiln atmosphere, giving rise to FeO, Fe_2_O_3_, or Fe_3_O_4_ [23]. This is evident in Figure 1, in which it can be seen that I, the Fe^0^ additive, tends to gain a lot of mass in an air atmosphere (>30%) just after a very exothermic peak with its maximum around 600 °C (Figure 1a).

However, its mass changes oscillate in a very narrow range in an inert argon atmosphere (Figure 1b), as also occurs with H (i.e., Fe_2_O_3_) both in air and Ar (Figure 1c,d), which in no case would be an indicator of any type of oxidizing reaction, and if it were, its contribution would be almost negligible. With respect to the remaining samples containing native iron, the oxidation of the iron has not been great enough to compensate for the loss of mass that occurs due to thermal alteration of the remaining components. As described previously [11], thermal analysis suggests the main mass loss would be connected with the dehydroxylation of kaolin between 400–600 °C, releasing H_2_O(g); the oxidation of organic matter from cork between 250–500 °C, emitting CO_2_, CO and H_2_O(g); and the dissociation of sodium carbonate, releasing CO_2_ above 850 °C.

**Figure 2:**
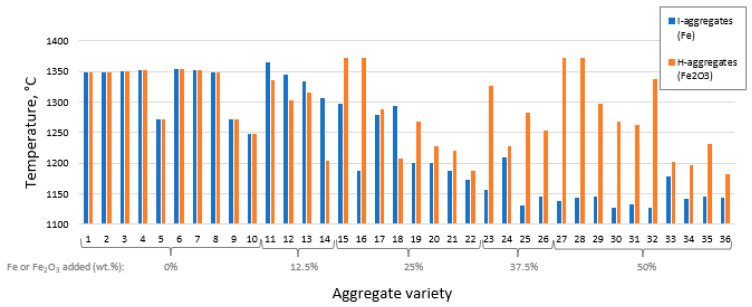
Firing temperature applied for each aggregate variety differentiating the iron additive used in the starting mixture.

In addition, the firing temperature has been significantly affected by the incorporation of the additives. As shown in Figure 2, if no iron phase is included (mixtures 1 to 10), no decrease in working temperature is achieved unless the maximum planned sodium carbonate is added (mixtures 5, 9 and 10 of I and H in Table 1), which would be as expected, given that Na_2_CO_3_ is a flux.

However, the addition of iron in the blends has led to significant changes. In the case of zero-valent iron powder, I, the working temperature generally tends to decrease as its proportion in the mixture increases, and is apparently not affected by changes in the proportions of the other two additives, C and N. On the other hand, although the addition of H has also favored a general decrease in working temperature (though it is less pronounced than for I), certain mixtures are observed with temperatures above 1300 °C and even 1350 °C, specifically, mixtures H15, H16, H23, H27, H28 and H32 (Figure 2). In the first five, the sum of C + N is ≤ 2.5%, while the last one has no N. This indicates that the effect of the starting percentage of H on temperature is much more affected by the other additives than in the case of I. This is likely due to a redox reaction, which will be explained in Section 3.4.

### 3.2. Bloating Index

**Figure 3:**
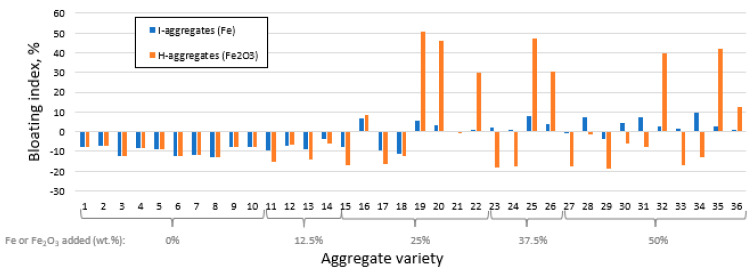
Bloating index for each aggregate variety differentiating the iron additive used in the starting mixture.

The *BI* results obtained for each mixture are shown in Figure 3.

As shown, the expansion process is closely linked to high proportions of iron (≥25 wt.%). In any case, the volumetric increase when adding I is notably lower than H, so that in the former there is no aggregate variety with *BI* higher than 10%, while in the latter there are aggregates with *BI* between 30 and 50% (varieties H19, H20, H22, H25, H26, H32 and H35). Conversely, although the addition of native iron produces smaller magnitudes of *BI*, it does give positive values of this property in a greater number of samples. In fact, several mixtures with H have experienced significant volumetric shrinkage (eleven with negative *BI* values greater than 10%), an aspect that is not observed when I is added. This shows that the capacity to undergo variations in aggregate size is much more noticeable when H (i.e., Fe^3+^) is added than with I (i.e., Fe^0^), which could be related to the development of a mineral body with a most adequate viscosity for trapping gas during firing [3].

**Figure 4:**
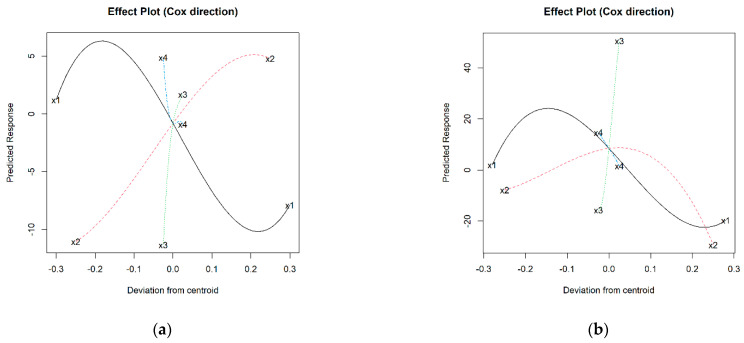
Effect plots of bloating index for: (**a**) I-aggregates and (**b**) H-aggregates; where: x1 = % K (solid black line); x2 = % I or H (dashed red line); x3 = % C (dotted green line); x4 =% N (dashed blue line).

To understand these variations, it is necessary to study the interaction with the other components involved in the mixture. Considering the results of the 36 starting mixtures, a cubic model has been obtained for each iron phase (see Tables S3 and S4 in the Supplementary Materials), from which the effect graphs in Figure 4 are plotted.

As indicated above, it is confirmed that aggregate expansion requires increasing percentages of I in the mixture, something that also occurs with H up to percentages of approx. 35–40 wt.%, above which, the effect is the opposite. Considering that the range of kaolin oscillates between 100% (I1 and H1) and 40% (I36 and H36) and that therefore its centroid is 70%, generally the *BI* is higher when K content is around 45–60 wt.%, tending to decrease notably for higher values. This trend would be somewhat stopped from approximately 85–90 wt.% of K, although insufficiently so as to be comparable to values recorded for low proportions of K. In the case of N, the flux, the graphs in Figure 4 suggest that its addition did not favor bloating during firing; rather, the opposite. This aspect contrasts with the role of C, whose increasing trend and steep slope denote that it promotes expansion (Figure 4).

**Figure 5:**
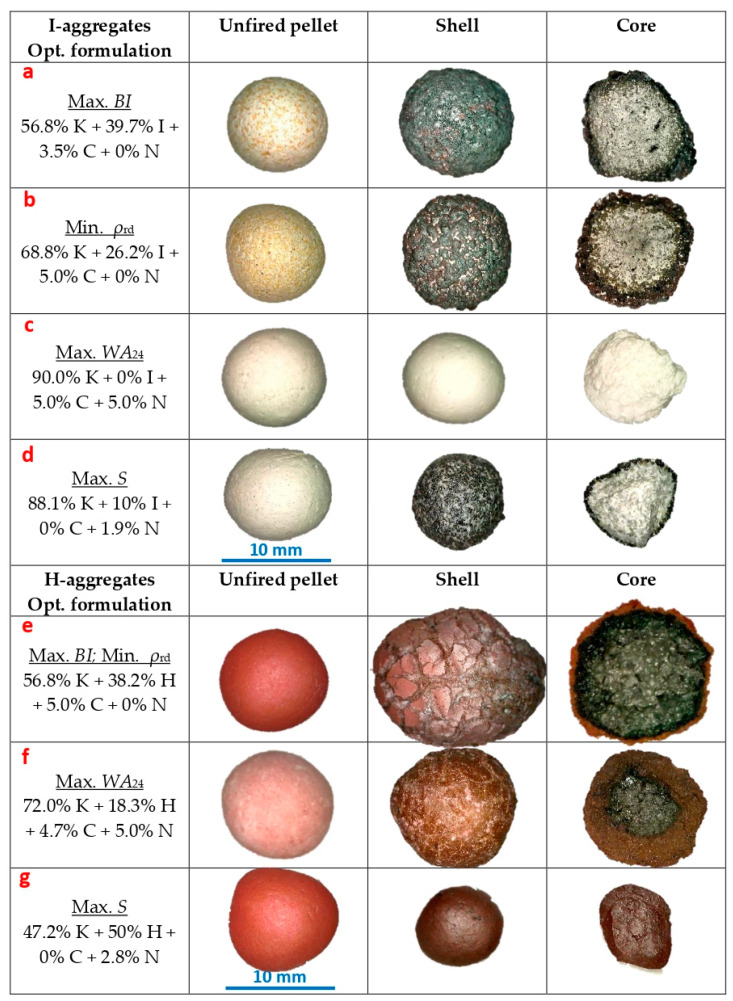
Optimal formulations and aggregates obtained from them, differentiating between the Fe^0^ (I-aggregates) and Fe^3+^ (H-aggregates) series.

Once the models have been fitted, they can be used to find mixtures with the maximum bloating index value using numerical methods. This analysis was performed using the statistical software Statgraphics 19. As is the case for numerical procedures, the starting point may affect whether a global or local optimum is located; several starting points have been used. Thus, the statistical analysis has estimated that to obtain the maximum bloating index value, the optimum formulations would be 56.8% K + 39.7% I + 3.5% C + 0% N in the case of the mixtures with native iron, and 56.8% K + 38.2% H + 5.0% C + 0% N for the samples with added Fe^3+^ (some representative specimens obtained from these are shown in Figure 5a,e).

Both formulations are very similar, indicating that although the effect of each type of iron is of a different magnitude, the trend is similar. The bloating phenomenon is closely linked to the formation of pores and the consequent decrease in density, aspects that will be discussed in Section 3.4.

### 3.3. Density

**Figure 6:**
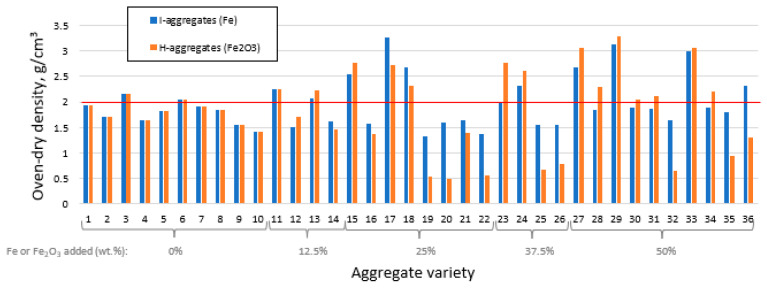
Oven-dry density for each aggregate variety differentiating the iron additive used in the starting mixture.

Figure 6 shows the density results (in this case *ρ*_rd_) of the aggregates obtained from the initial mixtures. Seventeen of the thirty-six varieties of both I and H have values above 2.00 g/cm^3^, which means that they cannot be categorized as lightweight aggregates according to the EN-13055-1 [24] standard.

In general, these high densities are more pronounced in those mixtures with ≥25 wt.% of iron (either I or H) that have undergone shrinkage during firing (Figure 3). The opposite occurs in those mixtures that presented higher *BI* values when fired, which are now the ones that exhibit the lowest densities, highlighting the mixtures 19, 20, 22, 25, 26, and 32 (Table 1), both for I and H. Although the trend is similar between the H and I mixtures, the decrease in specimen density is much more marked with the former than with the latter, specifically when the density is <2.00 g/cm^3^ (Figure 6).

The formation of pores related to greater bloating in the aggregates containing H, which would probably be the main factor leading to the differences in density with respect to specimens containing I. However, the type of iron added is also relevant. In stoichiometric terms, 100 g of I contains 100 g of Fe, while 100 g of H contain 69.9 g of Fe. Considering the high atomic mass and density of iron, this means that, for equal porosity, bloating and percentage of additive incorporated, the aggregates with I are heavier because the mineral matrix contains about 30% more iron atoms than the same mixture for H.

**Figure 7:**
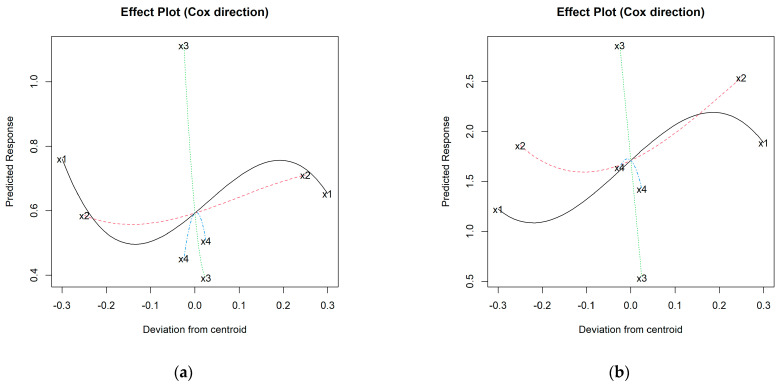
Effect plots of oven-dry density for: (**a**) I-aggregates and (**b**) H-aggregates; where: x1 = % K (solid black line); x2 = % I or H (dashed red line); x3 = % C (dotted green line); x4 =% N (dashed blue line).

As shown in Tables S3 and S4 (Supplementary Material), the best fitting model for *ρ*_rd_ was the special cubic for both the I- and H-aggregates (in the case of the former, a logarithmic transformation was necessary after diagnosis without transformation). The effect plots obtained are shown in Figure 7.

The trajectories for K and C are rather opposite to those discussed in Section 3.2 for the bloating index. This makes sense, since it is expected that the higher the bloating index, the lighter the aggregate structure. In the case of N, the density of the I-aggregates (Figure 7a) tends to increase up to about 2.5 wt.%, then decreases the magnitude of this property, although it still maintains values above those of the mixture without N. This trajectory is similar for H (Figure 7b), so the density is higher at the centroid (2.5 wt.% N) and when moving from it, this property decreases. In any case, the effect of C is greater than that of N.

Regarding I and H, up to about 10–15 wt.% iron, the density decreases slightly and then, once this percentage is exceeded, it tends to rise, so that the density would exceed the values of the iron-free mixtures in percentages close to and above the centroid (~20 and ~30 wt.% of I and H, respectively). This phenomenon is more marked when H is added than with I (Figure 7). Such an increase would be connected to the high density of the iron components, which, as described above, would favor the formation of a heavier mineral matrix. On the contrary, iron also tends to promote bloating (Figure 4) and pore formation (something to be discussed later), thus reducing the density. As can be seen in Figure 6, where the mixtures with the highest and lowest density are those with ≥ 25 wt.% of I and H, iron promotes two opposite effects at once. In this regard, if the density of the aggregate is to be reduced sufficiently, the generation of pores and the associated volumetric expansion must be large enough to counteract the increase in density of the mineral matrix due to the addition of iron. Thus, the estimated optimum mixture to obtain the minimum density is the same as the one indicated for the maximum *BI* in the case of H-aggregates, while for the mixtures with native iron, the estimated optimum is 68.8% K + 26.2% I + 5.0% C + 0% N (Figure 5b,e).

### 3.4. The Role of Fe^0^ and Fe^3+^ in Expanded Clay Aggregate Formation

**Figure 8:**
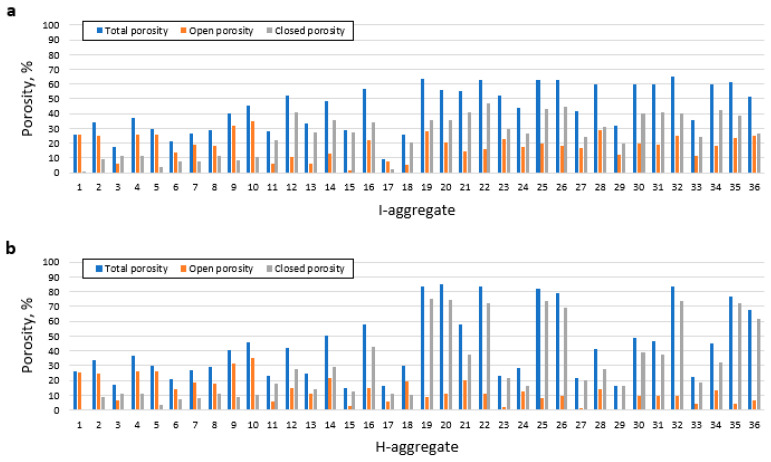
Porosity developed in the aggregates of the Fe^0^ study (**a**) and the Fe^3+^ study (**b**).

The porosity results of Tables S1 and S2 are depicted in Figure 8.

The addition of iron, either in elemental, zero-valent, form or as an oxide, has favored important changes in porosity. As was the case with *BI* (Figure 3), this impact is much more marked when H, i.e., Fe_2_O_3_, is used as an additive, highlighting mainly the development of total porosities above 60%, 70% and even 80% in varieties H19, H20, H22, H25, H26, H32, H35 and H36, which coincide with the types of aggregate that expanded the most as discussed above. These same varieties for the I-aggregates would also be among the most porous, although, in their case, the top is at 65% total porosity (aggregate I32). The development of porosity seems, therefore, to be favored by the presence of high concentrations of iron (≥25 wt.%) and organic carbon.

In addition to the total porosity, it is necessary to analyze whether it is open or closed, since the development of closed porosity to the detriment of open porosity is an important characteristic in the development of lightweight aggregates [3,25]. In general, a predominance of open porosity is observed in the iron-free mixtures (formulations 1 to 10 in both I and H in Figure 8). This trend changes when iron is incorporated in the mixtures, leading to sintered specimens generally showing a predominance of closed porosity over open porosity, which is especially noticeable in those varieties that are more expanded and porous.

Since the development of porosity, and especially closed porosity, is the factor that is closely linked to bloating and lightness of the aggregate, it is necessary to understand what reactions might be involved. The formation of Fe^2+^ (FeO when combined with oxygen in mineral samples) would be particularly relevant, since it is an iron phase that facilitates the development of a viscosity suitable for trapping gases, forming pores and leading to bloating [3,5,8,26,27,28]. In expanded aggregates, generally the predominant reactions will be oxidizing in the shell, while in the core they are reducing. Taking this into account, in those lightweight aggregates to which H has been added, this clear differentiation between shell and core has only been observed when C has also been added to the mixture. Thus, those varieties that present added hematite, but not C, are red both inside and outside (e.g., Figure 5g), showing that the necessary reducing conditions for the development of FeO have not been produced. This contrasts with those aggregates that do contain both C and H, which are characterized by a black core (typical of FeO formation) surrounded by a reddish shell rich in unreduced Fe_2_O_3_ (e.g., Figure 5e,f). This is because the presence of organic carbon through the addition of cork powder entails rapid consumption of a good part of the oxygen inside the aggregate to react with the carbon. This combustion would therefore be incomplete, generating CO, which facilitates the reduction of Fe_2_O_3_ to FeO [8,27]. In turn, if part of the carbon remained unburned (not oxidized), it could also act as a reducing agent for iron [5]. From these reactions, in addition to the CO indicated above, CO_2_ and O_2_ would also be released, part of which (along with the gas released from the other components) could be retained in the mineral matrix to generate the pores of the aggregate [3,5,26,28]. 

In the case of aggregates with the native iron additive, I, the mechanism that has led to the formation of pores is different, since in this case the formation of FeO in the core does not require a reduction, but an oxidation. As was the case for the other iron phase, it has been observed that the addition of C favors aggregate expansion and increased lightness. This seems contradictory, since the CO formed would be reductive and in no case would lead to the formation of FeO directly from Fe. However, the combustion of C, apart from CO, also generates H_2_O(g), which could act as an oxidant according to the following reaction:

Fe + H_2_O(g) → FeO + H_2_ (1)

According to Equation (1), the water vapor generated from the burning of organic carbon could favor the formation of the required FeO, as well as the release of H_2_ that could be retained to form pores. This hypothesis seems to be well supported by observations of the structure and coloration of the specimens. In general, the I-aggregates show a very dark coloration in the shell (Figure 5a,b,d), which would indicate that FeO formation has taken place there, especially linked to the atmosphere of the kiln, which is rich in O_2_, as air is present. Some specimens even show a certain reddish coloration, due to the complete oxidation of Fe to Fe_2_O_3_ (Figure 5a,b). On the contrary, the core is lighter, with grayish tones, indicating that the formation of FeO in this case has been limited, which would explain why the volumetric changes and the porosity of the aggregates with I are much less marked than those with H. It is noticeable that this coloration of the core is even lighter in the specimens without C (Figure 5d), which are denser and have suffered shrinkage, which would be linked to a lower formation of FeO. Despite this, although to a lesser extent, these varieties have also developed pores. This could be explained because the dehydroxylation of kaolin phyllosilicates would also release H_2_O(g) [23], leading to the oxidation of part of the Fe to FeO, although this could be insufficient for expansion and would require the addition of C to promote it. Similarly, previous oxidation of the iron during wet maceration of the dough prior to extrusion should not be ruled out, as pointed out by the appearance of yellowish tones with some reddish stippling in the pellets with Fe before firing (Figure 5a,b). If this has occurred, the Fe_2_O_3_ developed at this stage could be reduced by C according to the mechanism explained above for the H-aggregates.

The redox changes both for the I- and H-aggregates are ratified through a Rietveld mineralogical analysis of two samples (Table 3).

In the case of the aggregate to which I was added, most of the Fe has disappeared, remaining less than 1 wt.%. However, 7.5 wt.% hematite (Fe_2_O_3_) and 5.9 wt.% hercinite (Fe^2+^Al_2_O_4_) have formed, indicating complete oxidation of part of the native iron for the former (in the aggregate’s shell primarily) and partial oxidation for the latter (most likely in the core). Regarding the selected H-aggregate, it presents 5.8 wt.% of hematite (presumably linked to the reddish shell), which denotes that part of the added Fe_2_O_3_ has not undergone any redox change. However, it also contains 1.5 wt.% of hercinite (Fe^2+^Al_2_O_4_), suggesting that Fe_2_O_3_ has been reduced, probably in the core according to its black color (Figure 5). In any case, the crystalline iron content both in the I- and H-aggregate specimens is very low, so it would be mainly integrated in the amorphous phase (majority phase in both cases).

These findings are important not only from the point of view of the investigation of iron, but also of the organic components, which would not only act as reducers of oxidized species (something that was already known), but could also act at the same time as oxidizers of reduced species, which gives them an essential role in the formation of lightweight aggregates.

### 3.5. Water Absorption and Crushing Strength

**Figure 9:**
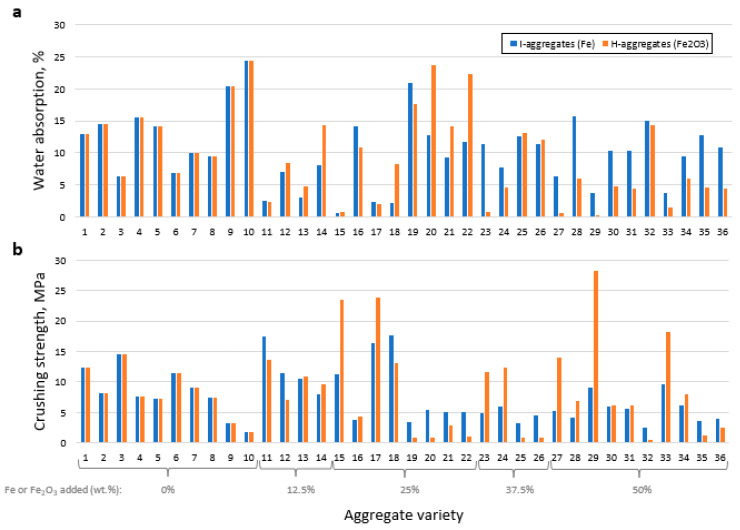
Water absorption (**a**) and crushing strength (**b**) for each aggregate variety, differentiating the iron additive used in the starting mixture.

The results of water absorption and crushing strength are shown in Figure 9.

Regarding the former, mixtures 9 and 10 present values above 20% of *WA*_24_, without the need to incorporate I and H (Table 1). However, these two formulations did present high contents of C and N, which, after decomposition, have favored the development of open porosity (Figure 8). Among the varieties with iron that show water absorption values > 20%, I-19, H-20 and H-22 stand out (Figure 9a); their formulations are characterized by containing 25 wt.% of iron and 5 wt.% of C (Table 1). 

**Figure 10:**
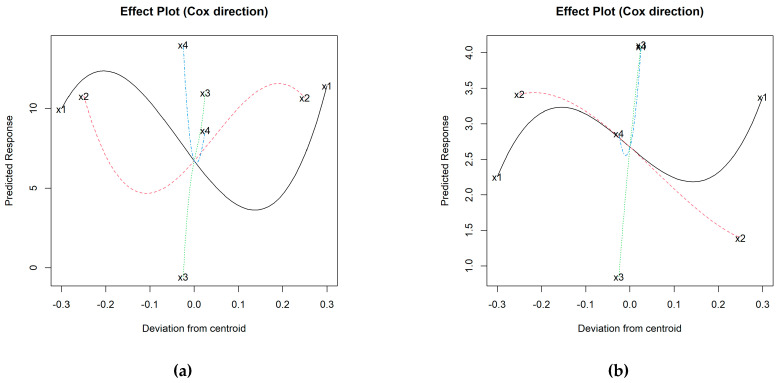
Effect plots of water absorption for: (**a**) I-aggregates and (**b**) H-aggregates; where: x1 = % K (solid black line); x2 = % I or H (dashed red line); x3 = % C (dotted green line); x4 =% N (dashed blue line).

Statistical analysis has revealed that the best fitting models are the cubic and special cubic ones for I and H, respectively; square root transformation was required for the latter to ensure the positivity of the model estimates (Tables S3 and S4). The results in Figure 10 indeed show that the C content significantly favors the increase in water absorption.

For the rest of the variables, the behavior is more complex. According to the N trajectories in the effect plots, the minimum *WA*_24_ value is recorded at the centroid (2.5 wt.% N) for the I-aggregates and around 2 wt.% N for the H ones, increasing the absorption progressively for both higher and lower N percentages. If the centroid is again taken as a reference for the I-additive curve (25 wt.% of I), higher percentages tend to increase water absorption, with maximum values around 40–45 wt.% of I. On the other hand, the minimum absorption value is associated with around 12–15 wt.% of I, while if iron is not added, absorption is comparable to that corresponding to 35 wt.% of I. On the other hand, the trajectory of H shows that the addition of Fe_2_O_3_ seems to negatively affect this property (Figure 10b). The kaolin content behaves similarly for both types of iron, whose maximum absorption values are around 50 and 100 wt.% of K, and minimum values at 40 wt.% of K in the H-aggregates and about 85 wt.% for both H- and I-aggregates. The models have estimated that the highest water absorptions would be associated with the mixtures 90% K + 0% I + 5% C + 5% N and 71.7% K + 18.6% H + 4.7% C + 5% N (some representative specimens are shown in Figure 5c,f).

The crushing strength results are shown in Figure 9b. The highest value is for aggregate H29 (28.2 MPa), followed by H15 and H17 (23.5 and 23.8 MPa, respectively), as well as I11, I17, I18 and H33, with values between 15 and 18 MPa. As shown in Figure 8, these aggregates are characterized by their very low porosity, which would be a determining factor in increasing crushing strength. This is evidenced by the fact that the less strong varieties are highly porous (e.g., H19, H20, H22 H25, H26 and H32). When comparing both types of iron additive, as was the case with *BI* and density, the range of results is wider when adding H, whose mixtures cover the maximum and minimum *S* values.

**Figure 11:**
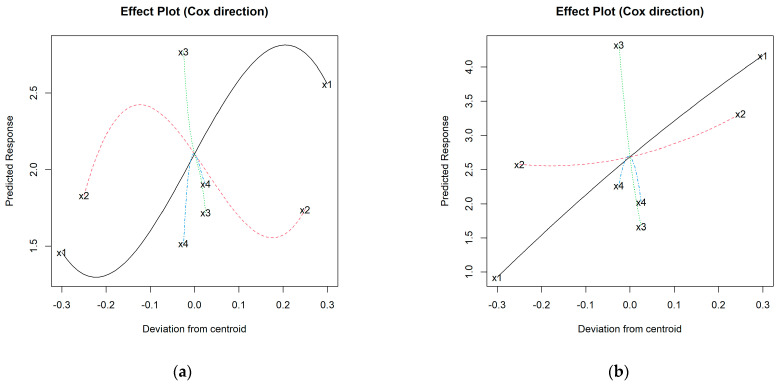
Effect plots of crushing strength for: (**a**) I-aggregates and (**b**) H-aggregates; where: x1 = % K (solid black line); x2 = % I or H (dashed red line); x3 = % C (dotted green line); x4 =% N (dashed blue line).

After an initial diagnosis of the model without transformation of *S*, the logarithm of *S* was required, through which the best-specified model was the cubic for additive I (Table S3). Figure 11a shows that the addition of C decreases the *S* results in this aggregate variety, while the addition of N would increase the crushing strength with maxima near the centroid (~2–2.5 wt.% N).

Also, an increase in K is generally linked to higher mechanical strength estimates, although with certain oscillations in the higher and lower proportions. The trajectory of the I-additive increases up to proportions of about 10–15 wt.% of native iron added. Thereafter, the trend is downward, although the *S* results would still be higher than those of mixtures without iron up to contents of approx. 30 wt.% I. Above this proportion, the mechanical strength would be reduced, with its minimum at approx. 40 wt.% of I. The formulation estimated to achieve the maximum value of *S* for the I-aggregates is: 88.1% K + 10% I + 0% C + 1.9% N.

In the case of additive H, the selected model was quadratic considering the square root of *S* (Table S4), whose maximum corresponded to the formulation: 47.2% K + 50% H + 0% C + 2.8% N. The effect plot for the H-aggregates (Figure 11b) suggests that strength increases with the proportion of K and up to approx. 3% N (maximum at around 2.5 wt.% N). The addition of C would decrease the mechanical strength, while H smoothly favors gains on this property. 

### 3.6. Characterization of Optimal Mixtures and Validation of Models

The main characteristics of the aggregates obtained from the optimum formulations (Figure 5) are shown in Table 4.

In the case of the I-aggregates, the optimum formulations for the maximum *BI*, minimum *ρ*_rd_ and maximum *WA*_24_ and *S* have led to the following results for these properties: 8.1%, 1.36 g/cm^3^, 24.5% and 20.1 MPa, respectively. These results are very close to those estimated using the mathematical models (*BI* = 9.8%, *ρ*_rd_ = 1.28 g/cm^3^, *WA*_24_ = 23.6% and *S* = 26.3 MPa). This trend is generally maintained for all the characterization data collected for each of the optimum aggregates (Table 4), so it can be stated that, in general, the models have been very useful for optimizing the desired properties, which agrees with the results obtained by the authors in a previous investigation using pyrite (FeS_2_) as an iron source [11].

Regarding the optimum aggregate varieties obtained from the H-mixtures, the differences between the data obtained experimentally are greater than those observed with the other iron phase: *BI* = 52.6% vs. 62.7% and *ρ*_rd_ = 0.57 g/cm^3^ vs. 0.32 g/cm^3^ in the mixture estimated for the maximum bloating index and the minimum density; *WA*_24_ = 16.4 vs. 27% in the mixture that sought the highest possible absorption capacity; *S* = 18 vs. 25.1 MPa in the formulation corresponding to the maximum crushing strength. In the case of the optimal aggregate for *BI* and *ρ*_rd_, despite the differences, the results obtained experimentally for these two properties improve the highest value of *BI* and are in the same order to the lowest recorded density with respect to the starting mixtures (*BI* = 50.4% for H19 in Figure 3 and *ρ*_rd_ = 0.50 g/cm^3^ for H20 in Figure 6). 

On the other hand, the water absorption and the mechanical strength of the H-aggregates designed to optimize these two properties not only provide low results with respect to the estimates, but are also much lower than the maximums registered in the starting mixtures, as can be seen in Figure 9 (*WA*_24_ of aggregate H10 = 24.5%, *S* of aggregate H29 = 28.2 MPa). However, the data in Table 4 for the optimal H-aggregates also show that these differences between the estimated and experimental values narrow significantly when the results of absorption and crushing strength are low. This can be observed in the optimum formulations of *BI* and *S*, whose estimated and experimental *WA*_24_ values are very similar. The same occurs in the optimal aggregates of *BI* and *WA*_24_, in which case, the mechanical strength data are very similar. Such observations show that although the models for the H-mixtures are not as accurate as those for I, they also have great potential to predict mixtures and results.

## 4. Conclusions

Based on the results obtained in this research, which mainly focused on the impact of Fe^0^ (here additive I) and Fe^3+^ (additive H) in the expansive process of kaolin-based lightweight aggregates, the following main conclusions are drawn:

- The process of expansion, pore generation and the associated decrease in density requires the addition of iron, such that the optimum mixtures of these properties presented between 25 and 40 wt.% of this additive, and also required the incorporation of organic carbon.
- The addition of H is linked to a greater volumetric expansion (max. 53%) than the use of I (max. 8%), suggesting that the formation of the FeO leading to bloating would require reducing and oxidizing conditions in the former and the latter, respectively. In this sense, the addition of organic carbon, C, seems to be crucial, since its incomplete thermal decomposition generates, on the one hand, CO, which would facilitate the reduction of Fe_2_O_3_ to FeO, while, on the other hand, the burning of C would release H_2_O(g) which, on the contrary, could intervene in the oxidation of native Fe to FeO.
- To obtain high mechanical strength, the addition of iron will be necessary, highlighting its combination with sodium carbonate (N) as a flux (especially in the range 2–2.5 wt.%) to obtain a more compact structure. Conversely, addition of organic carbon leads to a loss of mechanical strength. However, this component is crucial to facilitate open porosity formation, thus improving water absorption capacity.
- The experimental and model-estimated results are in good agreement, especially in the I-aggregates, which reinforces their application for further studies.

Overall, it is shown that the experimental and statistical protocols developed in this research with “ideal” raw materials could lay the groundwork for future research. In fact, this work is part of a larger project, in which the information gathered here will be used to manufacture lightweight aggregates with metalliferous waste as raw material. This will help to develop scalable procedures at industrial and commercial level.

## Figures and Tables

**Table 1:** Design of mixtures used to obtain the I-aggregates (Fe^0^ mix.) and the H-aggregates (Fe^3+^ mix).

**Fe^0^ mix.**	**I1**	**I2**	**I3**	**I4**	**I5**	**I6**	**I7**	**I8**	**I9**	**I10**	**I11**	**I12**
**K (%)**	100	97.5	97.5	95	95	95	95	92.5	92.5	90	85	82.5
**I (%)**	0	0	0	0	0	0	0	0	0	0	12.5	12.5
**C (%)**	0	2.5	0	5	0	2.5	2.5	5	2.5	5	1.25	3.75
**N (%)**	0	0	2.5	0	5	2.5	2.5	2.5	5	5	1.25	1.25
	**I13**	**I14**	**I15**	**I16**	**I17**	**I18**	**I19**	**I20**	**I21**	**I22**	**I23**	**I24**
K (%)	82.5	80	75	72.5	72.5	70	70	67.5	67.5	65	60	57.5
I (%)	12.5	12.5	25	25	25	25	25	25	25	25	37.5	37.5
C (%)	1.25	3.75	0	2.5	0	0	5	5	2.5	5	1.25	1.25
N (%)	3.75	3.75	0	0	2.5	5	0	2.5	5	5	1.25	3.75
	**I25**	**I26**	**I27**	**I28**	**I29**	**I30**	**I31**	**I32**	**I33**	**I34**	**I35**	**I36**
K (%)	57.5	55	50	47.5	47.5	45	45	45	45	42.5	42.5	40
I (%)	37.5	37.5	50	50	50	50	50	50	50	50	50	50
C (%)	3.75	3.75	0	2.5	0	2.5	2.5	5	0	2.5	5	5
N (%)	1.25	3.75	0	0	2.5	2.5	2.5	0	5	5	2.5	5
**Fe^3+^ mix.**	**H1**	**H2**	**H3**	**H4**	**H5**	**H6**	**H7**	**H8**	**H9**	**H10**	**H11**	**H12**
**K (%)**	100	97.5	97.5	95	95	95	95	92.5	92.5	90	85	82.5
**H (%)**	0	0	0	0	0	0	0	0	0	0	12.5	12.5
**C (%)**	0	2.5	0	5	0	2.5	2.5	5	2.5	5	1.25	3.75
**N (%)**	0	0	2.5	0	5	2.5	2.5	2.5	5	5	1.25	1.25
	**H13**	**H14**	**H15**	**H16**	**H17**	**H18**	**H19**	**H20**	**H21**	**H22**	**H23**	**H24**
K (%)	82.5	80	75	72.5	72.5	70	70	67.5	67.5	65	60	57.5
H (%)	12.5	12.5	25	25	25	25	25	25	25	25	37.5	37.5
C (%)	1.25	3.75	0	2.5	0	0	5	5	2.5	5	1.25	1.25
N (%)	3.75	3.75	0	0	2.5	5	0	2.5	5	5	1.25	3.75
	**H25**	**H26**	**H27**	**H28**	**H29**	**H30**	**H31**	**H32**	**H33**	**H34**	**H35**	**H36**
K (%)	57.5	55	50	47.5	47.5	45	45	45	45	42.5	42.5	40
H (%)	37.5	37.5	50	50	50	50	50	50	50	50	50	50
C (%)	3.75	3.75	0	2.5	0	2.5	2.5	5	0	2.5	5	5
N (%)	1.25	3.75	0	0	2.5	2.5	2.5	0	5	5	2.5	5

**Table 2:** Methods and equipment used in the characterization of the lightweight aggregates synthesized in this investigation.

Parameter	Method and Equipment (If Applicable)
Loss on ignition (LOI) during preheating and firing ^a^; %	Weight variation before and after firing 25 specimens in a Nannetti^®^ TOR-R 120-14 tubular rotary kiln
Bloating index (*BI*); %	Size variation before and after firing 25 specimens [18] in a Nannetti^®^ TOR-R 120-14 tubular rotary kiln
Loose bulk density (*ρ*_b_); g/cm^3^	Container filling [19]
Apparent density (*ρ*_a_); g/cm^3^	Water pycnometry [20]
Oven dry density (*ρ*_rd_); g/cm^3^	Water pycnometry [20]
24 h Water absorption (*WA*_24_); %	Water pycnometry [20]
Porosity: Total (*P*_T_) open (*P*_O_) and closed (*P*_C_) porosity; %	*P*_T_ = (1 − (*ρ*_rd_/*ρ*_solid_^b^)) × 100; *P*_O_ = (1 − (*ρ*_rd_/*ρ*_a_)) × 100; *P*_C_ = *P*_T_ − *P*_O_ [3,21]
Single aggregate crushing strength (*S*); MPa	Average value of 25 specimens/Nannetti^®^FM 96 press [3,22]

^a^ The LOI during firing embraces the complete firing process, in which the pellets pass through the whole kiln tube, highlighting the 10 min in middle at the set temperature. The LOI during preheating is that associated with the aggregates that remain for 2 min in the first third of the kiln tube, where the temperature is approx. 200–400 °C lower than that set. ^b^*ρ*_solid_ = 2.6 × [K/(K+(I or H)] + (6.5 or 5.2) × [(I or H)/(K + (I or H)].

**Table 3:** Mineralogical composition of two selected optimum aggregates.

Aggregate	Amorphous	Mullite	Quartz	Fe	Cristobalite	Hematite	Hercinite	Clinopyroxene
Max. *BI* 56.8% K + 39.7% I + 3.5% C + 0% N	57.5	17.7	2.9	0.9	7.6	7.5	5.9	
Max. *WA*_24_ 71.7% K + 18.6% H + 4.7% C + 5.0%	65.6	22.1	1.4		0.5	5.8	1.5	3.2

**Table 4:** Optimal formulations and comparison between experimentally determined and statistically model-estimated properties.

		Bloating Index (%)			Particle Density (g/cm^3^)			Water Absorption (%)			Crushing Strength (MPa)		
Optimum Formulation		Exp.	Est.	Dif.	Exp.	Est.	Dif.	Exp.	Est.	Dif.	Exp.	Est.	Dif.
**I-agg.**	**Max. *BI*** **56.8% K + 39.7% I + 3.5% C + 0% N**	8.1	9.8	1.7	1.67	1.47	−0.2	15	18.4	3.4	2.5	2.4	−0.1

	**Min. *ρ*_rd_** **68.8% K + 26.2% I + 5.0% C + 0% N**	5.3	7.4	2.1	1.36	1.28	−0.08	19.2	19.5	0.3	3.4	2.9	−0.5

	**Max. *WA*_24_** **90.0% K + 0% I + 5.0% C + 5.0% N**	−7.8	−8.2	−0.4	1.42	1.46	0.04	24.5	23.6	−0.9	1.7	2	0.3

	**Max. *S*** **88.1% K + 10% I + 0% C + 1.9% N**	−12.1	−14.9	−2.8	2.47	2.57	0.1	1.3	−0.9	−2.2	20.1	26.3	6.2

		**Bloating index (%)**			**Particle density (g/cm^3^)**			**Water absorption (%)**			**Crushing strength (MPa)**		
**Optimum formulation**		**Exp.**	**Est.**	**Dif.**	**Exp.**	**Est.**	**Dif.**	**Exp.**	**Est.**	**Dif.**	**Exp.**	**Est.**	**Dif.**
**H-agg.**	**Max. *BI*; Min. *ρ*_rd_** **56.8% K + 38.2% H + 5.0% C + 0% N**	52.6	62.7	10.1	0.57	0.32	−0.25	18.1	19.4	1.3	0.5	0.4	−0.1

	**Max. *WA*_24_** **71.7% K + 18.6% H + 4.7% C + 5.0% N**	10	17.8	7.8	0.83	0.66	−0.17	16.4	27	10.6	3.8	1.5	2.3

	**Max. *S*** **47.2% K + 50% H + 0% C + 2.8% N**	−19.5	−18.5	1	3.15	3.36	0.21	0.1	0.2	0.1	18	25.1	7.1


## Data Availability

The data presented in this study are available on request from the corresponding author.

## Supplementary Materials

The following supporting information can be downloaded at: https://www.mdpi.com/article/10.3390/ma16165623/s1, Table S1: Main characteristics of the aggregates obtained from the starting formulations with Fe; Table S2: Main characteristics of the aggregates obtained from the starting formulations with native Fe_2_O_3_; Table S3: Coefficients of the regression models and analysis of variance obtained for each key property in the I-aggregates; Table S4: Coefficients of the regression models and analysis of variance obtained for each key property in the H-aggregates.
