# Supplementary material for: Analyzing the Role of Fe0 and Fe3+ in the Formation of Expanded Clay Aggregates
Source: Materials (Basel). 2023 Aug 14;16(16):5623. doi: 10.3390/ma16165623 (PMC10456675; doi:10.3390/ma16165623)
Supplement: Supplementary file 1 [file materials-16-05623-s001.zip › Supplementary materials (Tables S1-S4)/Table S3.pdf]

Table S3. Coefficients of the regression models and analysis of variance obtained for each key property in the I-aggregates.

	BI (cubic)	*Log*(ρ_{rd}) (simplified cubic)	WA_{24} (cubic)	*Log(S)* (cubic)
**K**	-7.9	0.7	11.4	2.6
**I**	-87.3	0.2	-150.1	23.2
**C**	96379.8	380.3	138878.9	-14214.5
**N**	-133450.6	-311.9	-57065.2	13955.3
**cubic (K,I)**	-135.1	-	-242.2	29.7
**cubic (K,C)**	52874.8	132.9	75596.3	-7805.7
**cubic (K,N)**	-75887.4	-	-36779.0	7995.6
**cubic (I,C)**	61193.2	-	78500.0	-7564.6
**cubic (I,N)**	-70711.7	-294.2	-23467.4	7141.8
**cubic (C,N)**	-106160.5	-	-133725.6	18771.8
**K:I**	182.3	2.4	298.3	-44.4
**K:C**	-149079.6	-521.8	-214113.2	21979.4
**K:N**	208749.5	328.2	93267.1	-21896.0
**I:C**	-157619.5	-355.2	-216888.5	21720.6
**I:N**	205263.2	597.9	82269.7	-21241.7
**C:N**	47459.3	-	-71774.2	-512.3
**K:I:C**	117270.8	-	155493.2	-15384.3
**K:I:N**	-148615.3	-289.2	-62479.6	15421.8
**K:C:N**	-31595.8	-	24587.0	713.6
**I:C:N**	-22015.0	-322.0	33237.1	983.1
**F-statistic**	32.62	377.4	55.25	208.2
***p*-value**	2.158e-09	< 2.2e-16	3.731e-11	1.085e-15
R^2	0.9461	0.9927	0.9679	0.9914
