# Supplementary material for: Analyzing the Role of Fe0 and Fe3+ in the Formation of Expanded Clay Aggregates
Source: Materials (Basel). 2023 Aug 14;16(16):5623. doi: 10.3390/ma16165623 (PMC10456675; doi:10.3390/ma16165623)
Supplement: Supplementary file 1 [file materials-16-05623-s001.zip › Supplementary materials (Tables S1-S4)/Table S4.pdf]

Table S4. Coefficients of the regression models and analysis of variance obtained for each key property in the H-aggregates.

	BI (cubic)	ρ_{rd} (special cubic)	$\sqrt{WA_{24}}$ (special cubic)	$\sqrt{S}$ (quadratic)
**K**	-5.5	1.9	3.4	4.2
**H**	-549.7	2.9	7.3	6.1
**C**	-485251.5	-232.0	-3.0	420.4
**N**	-12382.6	-400.5	1431.2	-824.8
**cubic (K,H)**	-714.3	-	-	-
**cubic (K,C)**	-262661.4	-	-	-
**cubic (K,N)**	-13944.3	-	-	-
**cubic (H,C)**	-291698.1	-	-	-
**cubic (H,N)**	1241.9	-	-	-
**cubic (C,N)**	376049.2	-	-	-
**K:H**	1028.6	3.1	-18.2	-3.2
**K:C**	747087.1	242.0	13.6	-473.0
**K:N**	25609.1	422.4	-1497.9	844.8
**H:C**	773019.2	283.4	-148.7	-574.2
**H:N**	13952.7	450.8	-1644.1	905.1
**C:N**	660327.3	5287.4	-6628.9	538.4
**K:H:C**	-546064.2	-297.6	574.7	-
**K:H:N**	-15435.9	-61.1	335.0	-
**K:C:N**	-429441.4	5171.9	5981.4	-
**H:C:N**	-460743.9	-4876.0	4373.7	-
**F-statistic**	14.48	182.4	107.5	111.5
***p*-value**	8.942e-07	< 2.2e-16	< 2.2e-16	< 2.2e-16
**R^2**	0.8822	0.986	0.976	0.9684
